# Supplementary material for: Infections of the Xylella fastidiosa subsp. pauca Strain “De Donno” in Alfalfa (Medicago sativa) Elicits an Overactive Immune Response
Source: Plants (Basel). 2019 Sep 7;8(9):335. doi: 10.3390/plants8090335 (PMC6784145; doi:10.3390/plants8090335)
Supplement: Supplementary file 1 [file plants-08-00335-s001.zip › Supplementary files/Supplementary Figure 1, Symptoms on alfalfa.docx]

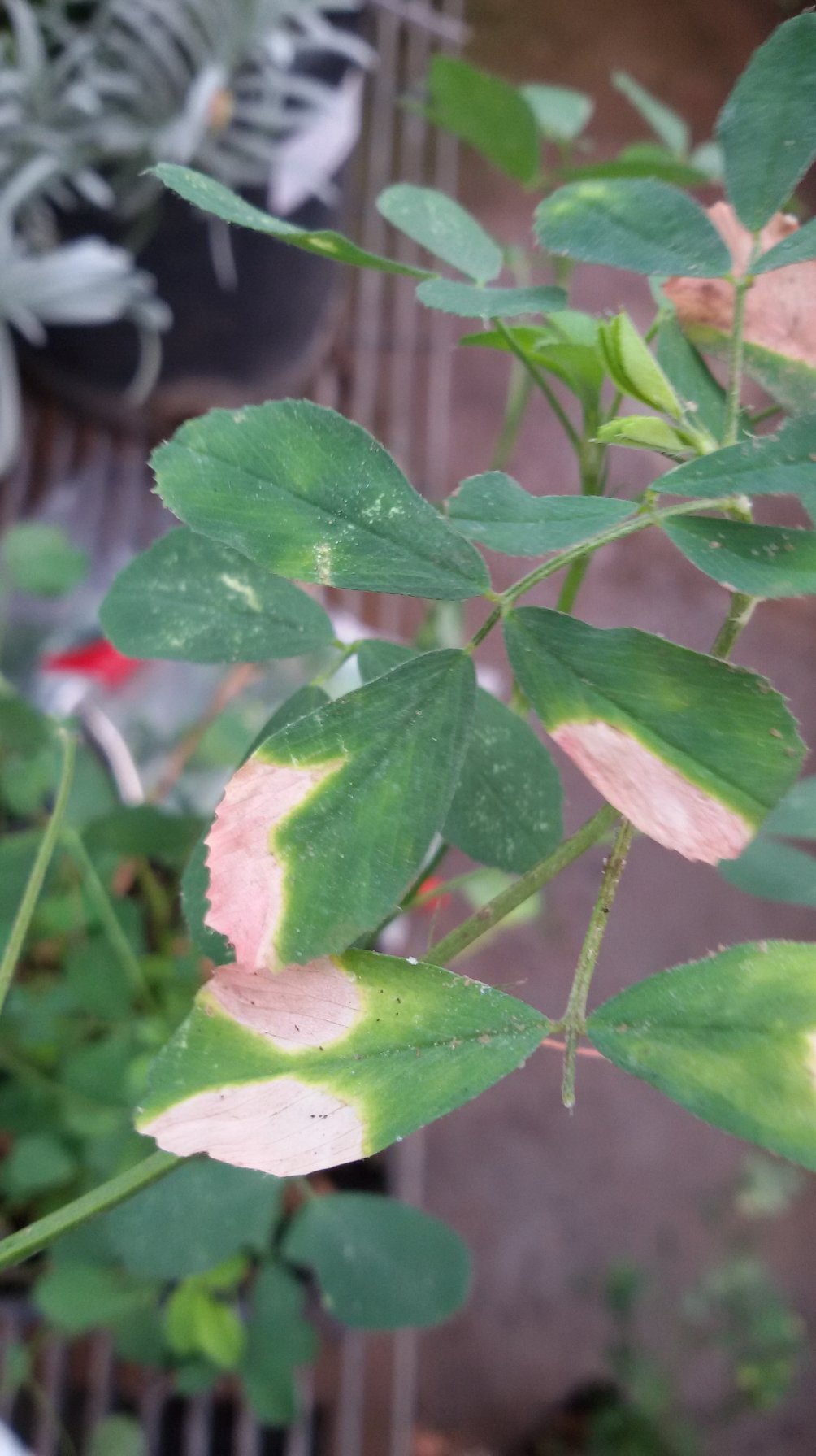


Supplementary Figure 1. Symptoms of leaf scorching recorded few months after the inoculation of the bacterium on Medicago sativa. Symptoms were detected regardless the status of the plants, either on plants testing positive and negative in qPCR assays , including the mock inoculated plants.
